# Post-doctoral research fellowship as a health policy and systems research capacity development intervention: a case of the CHESAI initiative

**DOI:** 10.1186/s12961-016-0159-3

**Published:** 2016-12-20

**Authors:** Martina Lembani, Gina Teddy, Dintle Molosiwa, Boroto Hwabamungu

**Affiliations:** 1 School of Public Health, University of the Western Cape, Robert Sobukwe Road, Private Bag X17, Bellville, 7535 Cape Town South Africa; 2 Health Policy and Systems Division, School of Public Health and Family Medicine, University of Cape Town, Anzio Road, Observatory, 7925 Cape Town South Africa

**Keywords:** Post-doctoral research fellowship, Health policy and systems research, Capacity development, Post-docs

## Abstract

**Background:**

Building capacity in health policy and systems research (HPSR), especially in low- and middle-income countries, remains a challenge. Various approaches have been suggested and implemented by scholars and institutions using various forms of capacity building to address challenges regarding HPSR development. The Collaboration for Health Systems Analysis and Innovation (CHESAI) – a collaborative effort between the Universities of Cape Town and the Western Cape Schools of Public Health – has employed a non-research based post-doctoral research fellowship (PDRF) as a way of building African capacity in the field of HPSR by recruiting four post-docs. In this paper, we (the four post-docs) explore whether a PDRF is a useful approach for capacity building for the field of HPSR using our CHESAI PDRF experiences.

**Methods:**

We used personal reflections of our written narratives providing detailed information regarding our engagement with CHESAI. The narratives were based on a question guide around our experiences through various activities and their impacts on our professional development. The data analysis process was highly iterative in nature, involving repeated meetings among the four post-docs to reflect, discuss and create themes that evolved from the discussions.

**Results:**

The CHESAI PDRF provided multiple spaces for our engagement and capacity development in the field of HPSR. These spaces provided us with a wide range of learning experiences, including teaching and research, policy networking, skills for academic writing, engaging practitioners, co-production and community dialogue. Our reflections suggest that institutions providing PDRF such as this are valuable if they provide environments endowed with adequate resources, good leadership and spaces for innovation. Further, the PDRFs need to be grounded in a community of HPSR practice, and provide opportunities for the post-docs to gain an in-depth understanding of the broader theoretical and methodological underpinnings of the field.

**Conclusion:**

The study concludes that PDRF is a useful approach to capacity building in HPSR, but it needs be embedded in a community of practice for fellows to benefit. More academic institutions in Africa need to adopt innovative and flexible support for emerging leaders, researchers and practitioners to strengthen our health systems.

## Background

### Need for capacity development in health policy and systems research (HPSR)

Efforts to build the field of HPSR have been explored by various scholars, and include examining ways to support local policy development and health systems strengthening, especially in low- and middle-income countries (LMICs) [1]. Some of the questions that have guided the discourse on capacity building for the field involve the emerging opportunities and challenges for the development of HPSR, the removal of structural barriers that inhibit development of the field, and the sufficient individual as well as organisational capacity to help develop the field of HPSR in Africa [1–3].

HPSR strives to develop an understanding of its interconnectedness to various elements of complex health systems and to collectively improve societal health outcomes through multi-disciplinary health research and adequate multiple health systems actors’ engagement in policy processes [4]. Encompassing four major domains, namely health systems, health systems development or strengthening, health policy and health policy analysis [4], HPSR is an emergent, trans-disciplinary field where multiple inputs from various disciplines and multiple research endeavours and techniques contribute to its very adaptive yet distinct characteristics [4]. Moreover, there is a great recognition of the contribution of HPSR to health systems strengthening and universal health coverage goals [3]. There is also an acknowledgement of HPSR’s contribution to the health systems building blocks, namely the people, service delivery, human resources, medicines and technologies, governance, information, and financing, as well as of the development of a good understanding of the interrelations between these building blocks [5].

While the growth and considerable role of the HPSR field in improving health systems is widely recognised, there are various challenges that constrain the field [2], as well as misunderstandings and challenges about its nature, purpose and scope [6]. A major challenge is human capacities in the field, particularly in LMICs [7]; this sentiment is echoed by other scholars such as Adam et al. [8]. They argue for greater efforts in HPSR capacity strengthening in LMIC institutions. While various competencies have been identified as essential for both the HPSR field and HPSR researchers [2], the challenge is building adequate capacity at the different levels of the system, including individual and organisational levels [9].

### Approaches to capacity development in HPSR

Although the need to strengthen human capacity in the field of HPSR is strongly advocated, there are uncertainties about the best approach to use [10]. The emerging nature of the HPSR field, as well as its complexity and questions around the approach to HPSR capacity development therefore necessitate a holistic and innovative approach to this goal. Potter and Brough [11] posit that a systemic capacity building approach is important if capacity building efforts are to yield better results. They advance several interrelated components that need to be addressed, including tools, skills, staff and infrastructure, as well as associated capacity levels at the individual and organisational levels [11]. It is argued by Green and Bennet [12] that a systems approach is necessary for adequate capacity development. A key challenge in this regard is how to create HPSR expertise while maintaining the field’s growth, uniqueness and complexity [1]. Essential to capacity strengthening is capacity assessment, which as argued by Le et al. [10], can also be a capacity development intervention.

In assessing HPSR capacity development forms, Bennett et al. [2] noted that short courses have largely been the main form of capacity development. They argued that, although the relevance of short courses cannot be underestimated, their overall contribution to developing HPSR interdisciplinary competencies was questionable. While acknowledging the contribution of these short courses to research capacity development, they advocated for investment in more comprehensive capacity development means such as graduate programmes and scholarships [2]. Post-doctoral research fellowships (PDRF) are one such capacity development measure. However, gaps still remain in the literature regarding how most effectively to build or strengthen capacity on HPSR through PDRF.

Capacity building needs in the HPSR field require not only adequate capacity strengthening strategies, but also higher education institutions with the necessary HPSR expertise and capacity [9]. In an analysis of capacity development strategies, Bennett et al. [13] identified strategies such as mentoring, research seminars, conferences, fellowships, internships, research grants, partnerships, short course training, networking, post-graduate training, and writing and publications support. Although the need to strengthen human capacity in the field of HPSR is greatly advocated for, there are uncertainties about the best approach to use [10].

### PDRF as a capacity development approach

While the strategies described above can have different implications at different levels and in different contexts, innovative fellowships are identified as beneficial for institutions and fellows. Historically, some disciplines, such as psychology, engineering and the biological sciences, have used PDRF as a form of advanced training to extend their graduates’ research competencies beyond graduate level training [14–17]. PDRFs are known to have been part of the European academia for centuries and later exported to the USA in the 1870s [18]. Today, more disciplines offer PDRF training to the point where it has almost become a de facto requirement before one can progress to a permanent academic or scientist position [15, 19]. Overall, the level of participation in PDRFs varies both by field of study and job-market characteristics, but with PDRF awards consistently being most prevalent in the biological sciences [18].

There is considerable potential mutual benefit for both institutions and the fellows to be derived through PDRFs appointments and engagement. Institutions award PDRFs for reasons such as host academic mentors’ need to realise full potential in research activity and accomplishments, as well as the need to attract and retain sufficiently qualified academic staff [20]. PDRFs generally provide an opportunity for the building up of scientific and technical human capital for the awarding academic institutions and organisations [21]. Post-docs, on the other hand, choose post-doctoral appointments for reasons including the benefits and opportunities for PDRFs, although engagement differs across places. There is evidence where, in some developed countries, fellows have been able to use a PDRF to establish networks and find employment [22–24]. On the other hand it has been reported that fellows in developing countries (although similar incidents reported elsewhere) have also experienced some negative effects of the PDRF including lack of committed mentorship [25].

PDRFs differ in how they are defined, their structure and the conditions governing the appointee [26]. It is most likely that the observed differences in PDRF awards are driven by specific goals and expectations of those awarding them. The structures and standards of PDRF can be project-based, mentor driven, appointee driven or teaching and research focused in the quest for inculcating competencies such as teaching, advanced knowledge of the specific field, and advanced research and professional skills throughout the related activities. Typical PDRF activities include independent development and conduct of research, development of grant proposals, manuscript submission, participation in existing research projects and field-specific professional activities, integration of research and practice, and provision of academic support.

A PDRF can be viewed as an essential component of a research career [27]. It is an opportunity for one to develop advanced research skills, while also learning the professional practices in a given field or discipline. At a broader level, PDRF training often invokes various workplace-based learning approaches, exposing appointees to tacit knowledge and technical know-how through participation in professional networks.

### The CHESAI PDRF

The University of Cape Town and the University of the Western Cape have, through the Collaboration for Health Systems Analysis and Innovation (CHESAI), undertaken to bridge the HPSR capacity gap in Africa by appointing four postdoctoral fellows (here forth referred to as post-docs) from Africa. In this paper, we examine the contribution of the CHESAI PDRF as a capacity development intervention in HPSR in a bid to fill some of the gaps in the capacity development literature. We reflect on our PDRF experiences to highlight the contribution of the CHESAI post-doctoral programme in developing, shaping and sharpening our understanding and knowledge of the HPSR field. The paper considers the broad question: In what ways can a PDRF foster capacity development in HPSR? A full description of the CHESAI PDRF in terms of its structure and activities is provided in the methodology section under Case study description.

## Methods

We used a reflective approach similar to Gibbs reflective cycle, which enabled us to draw narratives of our personal experiences as CHESAI post-docs [28]. This approach allowed for an in-depth analysis of our experiences in terms of competencies required for capacity development in HPSR, using multiple sources of evidence from our real-life learning context and experiences [29]. We used this approach in recognition and appreciation of the increasing interest in reflective practice “*as a way of developing complex understanding of practice that is helpful in an increasingly multifaceted and uncertain environment*” [30]. Working in HPSR, and in healthcare in particular, always point one to the interdependence of our world systems. Drawing on the elements of reflective practice was not only useful as a research strategy used for this paper, but we found reflective practice very relevant to our learning and in establishing ourselves as young HPSR researchers.

### Case study description

CHESAI is a 4-year (2012–2016) collaborative effort of the Schools of Public Health at the University of Cape Town and the University of the Western Cape, established to promote and extend the emerging field of HPSR in South Africa and Africa as a whole. With an explicit social science perspective, efforts mainly focus on conceptual and methodological development for the field through multi-sector engagement and across disciplines. The aims of CHESAI include capacity development in HPSR, building an intellectual hub for HPSR development and creating spaces for research-practice engagement. To realise these aims, CHESAI seeks to provide opportunities and an environment for deepening the HPSR knowledge base through variable activities, including supporting research activities, talking across disciplinary boundaries and facilitating analytical engagements across existing collaborative projects. The key activities include biannual writing retreats, bimonthly journal clubs, bimonthly meetings and annual invitations of HPSR senior researchers (Expert Residents) and practitioners (Practitioner Sabbaticals) from LMICs. In addition, CHESAI awarded four PDRF appointments to early career African researchers as a form of capacity development in HPSR. Each post-doc was awarded a 2-year fellowship at different times between 2014 and 2015.

The four post-docs originate from four different countries – Ghana, Malawi, Democratic Republic of Congo and Botswana. Of these, three are female and one male, with variable education backgrounds ranging from social policy and social work, health information systems, and international development studies to epidemiology and population health, thus capturing the multi-disciplinary perspective required in HPSR. However, all of their PhD studies were in the field of health, focusing in health financing, health information systems, HIV and aids service provision, and maternal and child healthcare services. In all cases, the focus was on policy implementation processes and outcomes. By April 2016, when the final reflection and writing of this paper began, all of the four post-docs had been engaged in the fellowship for a minimum period of 15 months. However, by June, at the time of submission of this manuscript, one had completed her 2-year appointment.

The postdoctoral fellowships (among other awards) are intended to strengthen the knowledge base in HPSR necessary to help build the field and thereby create equitable health systems in LMICs, especially in Africa. By providing us with opportunities for experiential learning, it is anticipated that the award will serve as a pathway for further academic and professional development, grounding us as emerging health policy and systems researchers. Immersed in a community of practice around HPSR, among other activities, we are expected to conduct research and produce academic papers on any of the CHESAI thematic areas, which include leveraging change in complex health systems, harnessing tacit and experiential knowledge to health system development, catalysing multi- and trans-disciplinary inquiry to support health systems development, and strengthening the research-practice interface. This is not to say that the CHESAI PDRF is research based – it is rather a very flexible appointment that recognises HPSR as an emerging field. Therefore, seeking to allow us to be a part of the development of the conceptual and methodological foundations of the field, we are mainly given the time and space to engage broadly in a community of practice in HPSR. The CHESAI community of practice consist of a group of health systems and policy researchers, practitioners and advocates working collaboratively to learn, explore and share experience to build the field of HPSR, develop concepts and frameworks, and leverage practices that strengthen health systems. We also participate in various existing projects, support teaching and student supervision, and the dissemination of ideas through conferences and seminars. Therefore, this makes it an interesting case to understand in what ways a PDRF can be a useful approach for capacity development in HPSR, using reflections of our post-doc experiences.

### The research process

The reflection process was very iterative in nature in that it involved multiple meetings where we discussed the research process following a systematic step-by-step process starting from data collection to analysis [31]. Similar to Gibbs reflective cycle [28], we began with an initial reflective group discussion on our experiences of the CHESAI programme overall, which led to the construction of a question guide for data collection. The guide consisted of questions coined around identifying the range of activities we were involved in, our experience of them, their relevance to us, what worked or did not work well for us, and what HPSR experiences or other skills we gained. We also reflected on the nature of the fellowship and how it encouraged or constrained learning, and on any lessons learnt on the PDRF approach as a capacity development tool. Each one of us then wrote a reflective narrative using these guiding questions reflecting on our experiences over the period of our engagement with the CHESAI PDRF. The process provided the flexibility of recounting experiences that were directly and indirectly related to the PDRF, capacity and competency development and the building of the field of HPSR in general. These narratives were informed by our personal and work diaries containing information on meeting proceedings and other information, reports (both verbal and written), deep reflections, observations, action learning processes, participation in CHESAI-related and external activities, evaluations and a range of other processes. These activities and sources of data informed the initial narratives and subsequent thematic analysis.

The next step was the iterative process of analysing the data. It involved a series of repetitive and recursive processes to capture new information and allow for rigour [32]. It started with each post-doc reading all four narratives to compare the similarities, differences and points of interests. This was followed by a group discussion where each post-doc explained their narratives and clarified points while identifying the highlights of their experience to the other post-docs and new information was generated and written down in our notebooks. We had a follow-up group discussion to collectively identify descriptive and analytical themes emerging from the narratives as well as to clarify contradictions and unclear points. This achieved two goals, (1) to identify our individual and collective experiences and (2) to cluster the key findings into themes. Excerpts from the broader descriptive themes were labelled and regrouped into analytical themes that provided a synthesis of key concepts discussed in the findings. Using the analytical themes as concepts of analysis, another round of individual reading of all four narratives was done and further validated collectively in relation to the research question, our expectations and CHESAI activities to inform the findings and discussions. This information was captured on flip charts and Post-it notes to enable categorisation of the issues and themes that were generated through these processes. Finally, a group discussion on the findings was held to allow us to interpret the data and give it meaning in relation to the literature on capacity development in HPSR [33–36].

### Limitations

Like most single case study research, the methodological strategy predominantly follows narratives to suit the nature of the enquiry and the small sample size. To achieve an in-depth and thick description of the phenomenon for wider understanding, various subjective processes were adopted with a bearing on the data collection and analysis process. The data is derived from participants’ context-specific reflection and perceptions of capacity development in HPSR. Like most qualitative research, it is subjective to participants’ frames of reference, interpretations and depiction of social experiences [33]. The dual role of the authors as the unit of analysis of the study also makes it highly subjective. Therefore, our personal reflections and contextual relevance to other settings may not be entirely applicable to PDRFs in general. The nature of this study is thus interpretative, relevant to its context and does not seek to suggest statistical generalisability. It is a qualitative research, with the appropriateness of the strategies derived from the nature of the topic explored [34].

Nevertheless, a systematic process was used to engage in the process of reflection and validation of the data, experiences and activities to allow for progressive assessment, and informed feedback among colleagues. Although the data is largely based on authors’ real-life experiences, the aim is to provide useful insights from our post-doc perspectives on how PDRFs can be a useful approach to capacity building [37]. This reflective approach demonstrates the experiential learning space at various levels as the capacity building process developed in commensurate ways among the individual post-docs, the CHESAI group and the affiliated organisations [38]. This form of reflective practice is not unique to this study but has also been used by the Consortium for Advanced Research Training in Africa on assessing African capacity building in health, whereas the experiential learning from the Malaria Capacity Development Consortium demonstrate the need to strengthen public health capacities for doctoral and post-doctoral programmes in Africa in general [39, 40].

A systematic triangulation of the data was supported by systematic group validation, member checking and capacity development literature to ensure credibility and trustworthiness of the study [41].

## Results

This section presents the findings that emanated from the analysis of narratives of our experiences with the CHESAI PDRF. Despite variations in the time periods of engagement with the project as presented in the Methods section, the post-docs’ experiences were fairly similar. Our thematic analysis identified three broad themes with associated sub-themes. These are summarised in Table 1 and each theme and sub-theme is described in detail thereafter.

**Table 1:** Main themes and sub-themes on the post-doc experiences on CHESAI

Main theme	Sub-themes
Format of the post-doctoral fellowship	• Expectation versus reality • Sense of direction • Trusting the process
Support structures	• Space for reflection • Mentorship • Practical experience • Work resources and environment
Learning and professional skills development	• Theoretical grounding in HPSR • Networking • Teaching and student supervision skills

### Format of the PDRF

The flexible, non-structured and emergent nature of the CHESAI PDRF created both opportunities and challenges for the post-docs. Regarding opportunities, this approach offered room for exposure to a wide range of activities within CHESAI and beyond through participation in various HPSR-related initiatives. The non-structured nature of the fellowship made it difficult for us with regards to the sense of direction, managing expectations versus reality, and trusting the process.

### Expectations versus reality

As with other conventional post-doc fellowships where participants are expected to produce certain outputs such as publications per quarter or coordinating a particular project, we joined the fellowship with such expectations. However, this was not the case in the CHESAI fellowship, and this scenario created frustration among us due to the mismatch between our expectations and the reality of the fellowship, which focused on exposing us to the field of HPSR through various activities that took most of our time instead of focusing on publication, as we had expected. The CHESAI PDRF goals were more geared towards experiential learning for HPSR rather than fixed on one specific activity. Although this frustration did not last forever, it was nonetheless a major issue.

“*These projects and workshops are generally a good learning opportunity in terms of exposure to new concepts, networking and the overall participation in project activities. However, these projects can be disruptive as one loses concentration on one of the PDRF key output (at least for me): publication.*” (Post-doc B)

### Sense of direction

The flexibility and emergent format of the PDRF meant that the we did not have a clear sense of direction about what exactly we were supposed to achieve over the fellowship period. Although we were encouraged to publish work from our PhDs using skills gained in the HPSR field, there were no specific targets attached. Most of the time was dedicated to participation in different activities to expose us to HPSR work as much as possible. This is because the CHESAI PDRF incorporates strong apprenticeship elements where one learns on the job through the various platforms of the CHESAI activities. However, this added to the frustration, as one of the postdocs noted, as she felt as if she had not achieved anything after several months of commitment into the fellowship:

“*As much as the CHESAI PDRF is an emerging process, having clear targets may be helpful for fellows to work towards and to have clear commitments to achieving them. The absence of this can be frustrating because I personally constantly felt like I have achieved nothing at all in the process until I take time to reflect.*” (Post-doc A)

### Trusting the process

Due to this unclear sense of direction, it was hard for us to trust the process, especially in the early days of our engagement. Therefore, navigating through the system of the CHESAI PDRF was a challenging experience. Sometimes, we did not understand why we were given certain assignments, as these seemed to be not in line with our expectations. In some cases, others felt pressured to perform in certain activities in the initial stages when we did not fully understand what we were expected to do. For instance, at the first writing retreat, one of us recounted her experiences where she felt everyone else knew what they were doing, while she was not quite sure how to approach the whole writing retreat concept as she was experiencing it for the first time, and this created some anxiety:

“*At the first retreat, I felt pressured and very unsure; it was not quite clear for me what the modus operandi was…. To begin with, I do not think I went for the retreat knowing exactly how I was going to spend my time there…*” (Post-doc D)

However, this was not necessarily the case for all of us. One post-doc found her first writing retreat experience very rewarding as she was able to interact with other senior CHESAI colleagues who assisted her to use her time more effectively. However, this required individual initiative because no one would know that someone was struggling with the process:

“*The first retreat I participated in afforded me the opportunity to start writing publication papers from my doctoral thesis with assistance from one CHESAI member whom I requested to help identify areas in my thesis for paper writing and which journals were suitable for my papers.*” (Post-doc C)

In addition to these issues, we also found it a challenging experience to understand the language used in communicating in the field of HPSR, which required some adjustment on our part at the beginning of the fellowship. Sometimes, we felt lost during discussions and could not engage meaningfully:

“*Part of my initial role as a participant at my first journal club was to collate notes of the proceedings and report back both internally and externally. I found* [by reading through the previous journal club notes] *immediately that there is a core HPSR ‘language’ that is also part-and-parcel of HPSR features.*” (Post-doc D)

The need to quickly tune in to understand the HPSR language and be able to engage meaningfully with the CHESAI team and other HPSR players beyond was crucial. This was possible through reading materials that were provided by our supervisors and through continued engagement. With time, we gradually felt integrated into the CHESAI team, and started trusting the process, as we began to understand and appreciate the HPSR field. Notwithstanding all these initial pressures, we believe this was an important experience and probably a necessary process for us to locate ourselves within the bigger system, through self-awareness and discovery, participation and reflections. Once we achieved this, it was much easier for us to appreciate and utilise the space created for us for effective learning through the various support structures at our disposal:

“*CHESAI’s free open-ended approach is great. I would say that it is the most appropriate approach given the nature of HPSR and the background of the PDRF.*” (Post-doc B)

Overall, as time went on, there was a general change in our thinking and understanding of our engagement. This mainly emanates from the fact that CHESAI understands HPSR capacity development as something that entails developing new understanding and gaining confidence in the field, as well as developing new ways of seeing and doing things. This is only possible by engaging with others through the process in collaboration and networking.

### Support structures

One outstanding feature of the CHESAI post-doctoral fellowship is its support structures and enabling environment that facilitated effective learning for us in the emerging field of HPSR. The support structures include the space for reflection, mentorship, practical experiences, and work resources and environment.

#### Space for reflection

The CHESAI initiative, through its regular activities such as the writing retreats, bimonthly meetings and journal clubs, provided spaces for us to reflect and develop skills in different areas within the field of HPSR. We were given the opportunity to contribute in such fora in various ways, and sometimes worked in teams with colleagues to accomplish specific tasks. These spaces were very critical for us to develop a better understanding of the HPSR field. During the journal clubs, retreats and bimonthly meetings, specific topics or papers were discussed in detail to allow deep reflections on methodological or conceptual issues:

“*As a PDRF, engaging in the journal clubs was an eye-opener because it created a safe space for researchers and practitioners of different units and organisations to critically assess and reflect on their own practices in-country and beyond… This has led me to deepen my knowledge on HPSR…..It has been an amazing learning curve…*” (Post-doc A)

Overall, we found such spaces useful for reflection on our work, and the opportunity to exchange knowledge with other CHESAI members and other practitioners a positive developmental space.

#### Mentorship

Mentorship is one of the integral parts of PDRFs for support and guidance to fellows to enhance learning. As CHESAI post-docs we feel we received enough support and guidance from our supervisors in various aspects of our professional development. Due to the flexible nature of the fellowship, our mentors exposed us to various activities they deemed relevant to the attainment of HPSR experience. Their leadership was highly appreciated as they took their time to reflect on issues related to HPSR, including encouraging us to publish work from our doctoral theses from an HPSR perspective, collaborative writing in CHESAI thematic groups and project report writing. The various papers that we have been working on are listed in Table 2. The mentors included both the Project Investigators as the prime mentors and other CHESAI members who were in constant contact with us. All the CHESAI team members were very helpful and willing to support us wherever possible. Our prime mentors took their time to meet with us individually to discuss and reflect on our experiences as we progressed, which helped us to review and focus our work where necessary.

**Table 2:** List of publication, project reports and working papers by post-docs

1. Scoping the application of theory of change in public health research (Draft manuscript) 2. Stakeholder participation in implementation of information systems (IS) strategy in public hospitals in the Western Cape Province, South Africa (In press) 3. Towards a framework of stakeholder relations’ influence on the implementation of information systems (IS) strategy (In press) 4. Developing the concept and practice of district health learning sites. A scoping review of the literature (In progress) 5. Health outcomes among patients on antiretroviral therapy (ART) across service providers in Malawi (In press) 6. A mixed method exploration of access to and utilization of maternal health services among women in Palapye, Botswana (manuscript ready for submission) 7. Examining trust in low- and middle-income countries – key methodological approaches (In progress) 8. Health systems resilience: a systems analysis. A Case study of maternal health service provision in OR Tambo District, Eastern Cape, in the context of chronic poor health performance (Published report) 9. Supporting system resilience and improved maternal health service provision in Eastern Cape, South Africa’s context of chronic emergency: A systems dynamics analysis using group model building (In press) 10. Health service resilience in Yobe state, Nigeria in the context of the Boko Haram insurgency: a systems dynamics analysis using group model building. (Published manuscript) 11. Exploring workplace learning (WPL) to support health managers in the Western Cape Province. An option for leadership development in the health system (Project report) 12. Health sector development in Ghana. A historic overview (Full manuscript, to be submitted soon) 13. Does leadership make a difference? Assessment of district health leadership and maternal health performance of two rural district hospitals in South Africa (Full manuscript to be submitted soon) 14. Towards sustainable leadership and management development within the health system: lessons from a partnership for health leadership and management capacity development in South Africa (Draft manuscript) 15. Policy and implementation gap: a multi-country perspective (Full manuscript to be submitted soon) 16. The impact of power and politics on policy implementation and reform in developing countries: the complexities of implementing Ghana’s health insurance (Full manuscript, to be submitted soon) 17. Mitchells Plain & Klipfontien sub-structure – partnership for health leadership and management report (Project report) 18. Northern Tygerberg partnership for health leadership and management report (Project report) 19. “Embedded systems approaches to health policy and systems research”. In Applied Systems Thinking for Health Systems Research: A Methodological Handbook Edited by Don de Savigny, Karl Blanchet and Taghreed Adam. Maidenhead Berkshire: Open University Press; 2016

“*The support I get from my supervisors is incredible. They are always available for guidance in many areas that I need their support for. Their commitment is unmatched and this gives me energy to work hard and move forward in my career.*” (Post-doc C)

Through mentorship, the supervisors ensured that we worked towards the development of our professional career growth through the apprenticeship elements embedded in CHESAI.

#### Practical experience

A unique feature that we found in the CHESAI PDRF was its embeddedness in the community of practice. The CHESAI team is highly involved in collaborative projects at various levels of the health system. This created an opportunity for us to engage with practitioners and understand the practical application of HPSR and how to influence policy. This experience exposed us to a better understanding on how to engage with practitioners in a meaningful and productive manner. The co-production approach between researchers and practitioners is very powerful in HPSR, as it ensures better uptake of research findings:

“*Being part of a team of researchers working with the Western Cape Provincial Department of Health, I’ve had the opportunity to be part of a research process that allows us to make, reform and evaluate policies, make meaning of those policies and translate them into practice for frontline workers. I have experienced through these policy engagements, the value of strong collaboration and active engagement between academia and the practitioners…*” (Post-doc A)

#### Work resources and environment

The environment within which we operated in the CHESAI PDRF, with regards to material resources such as office space, access to libraries, computers, printing services conference attendance and all other work-related necessities, was highly valued by all of us. It created an enabling environment for our learning. In addition, the fellowship offered a reasonable stipend to allow us manage our day-to-day basic necessities. We also appreciated the support we got from other non-CHESAI members in the departments we were attached to. In both universities, we were well integrated into the systems and got involved in the various activities of the departments, such as departmental journal clubs, research activities, academic work, as well as leisure-related activities like end of the year gatherings and other special events taking place in the department:

“*I cannot imagine functioning as a post-doc fellow without the necessary material support: the office space and facilities that we have make things easy for our research activities. Moreover, the stipend that we get allows one to survive here in Cape Town.*” (Post-doc B)

### Learning and professional skills development

The CHESAI PDRF aims to provide professional development skills to emerging researchers in HPSR. Through involvement in various fora and activities, we acquired relevant skills in teaching and student supervision, theoretical grounding in HPSR and networking.

#### Teaching and student supervision skills

Teaching and student supervision is yet another set of critical skills one requires for academic appointments. Therefore, acquiring this skill was very beneficial for us. Some among our team did not have any experience in teaching prior to joining the fellowship. However, each of us narrated our experiences in how we acquired skills in teaching (including curriculum development and module marking in one case) and Masters student co-supervision with our mentors as well as Masters thesis examination. These skills are essential for our professional career life. However, we did not have an opportunity to independently supervise or co-supervise students among ourselves due to time constraints, as the remaining time on our post-doc (after acquiring the skills) could not allow us to take a student through to the end of their Masters thesis project. However, the experience gained was significant enough to enable us independently to supervise a Master thesis.

“*…The CHESAI PDRF experience is also allowing me to develop my academic profile and prowess through teaching and supervision of MPH students.*” (Post-doc B)

#### Theoretical grounding in HPSR

Through the various CHESAI activities, such as the journal clubs, bimonthly meetings, writing retreats, short courses and participation in conferences, we acquired theoretical understanding of HPSR. The University of the Western Cape winter school courses in particular were considered a major breakthrough in understanding the theoretical underpinnings of HPSR for us. These short courses run for one week each and are offered to build the field of HPSR and cover topics ranging from defining the key concepts, such as HPSR and complex health systems, to understanding frameworks for analysing complex health systems, generating and framing HPSR questions, study design, rigour and ethics among other issues.^1^

“*The two winter school courses on Introduction to Complex Health Systems and Introduction to HPSR were a great foundation for my understanding of what HPSR is all about and helped me to build my theoretical base for HPSR including the methodological approaches and its inter–disciplinary nature more concretely. These courses created much interest for me in the field. This experience further served to piece together all that I had learned in the journal clubs, bimonthly meetings and other fora.*” (Post-doc C)

#### Networking

Creating networks is yet another very significant aspect of professional career progression for wider opportunities and collaboration. However, we found it difficult to achieve this in the beginning due to lack of skills on how to approach other researchers and create effective networks. Nonetheless, with more exposure through working in various projects and participation in a variety of activities, such as the journal clubs, conferences, project meetings and short courses, as well as our interactions with Practitioner Sabbaticals and Expert Residents, we slowly began to build up our networks:

“*Thus the CHESAI activities have taken me through an invaluable learning curve of horning some basic understanding and skills of negotiation, networking and working with practitioners and researchers.*” (Post-doc D)

Generally, we feel that we were provided enough opportunities to network and collaborate for professional growth. This is critical for our future career development. For instance, each one of us went to at least three international conferences, three of us have collaborated with one of the resident experts who visited CHESAI and we are currently in the process of developing a joint research proposal. We hope that this network will be strengthened through working together in this project if our proposal is accepted by the funder.

However, despite all these diverse experiences, we still felt inadequate in some of the most critical professional development aspects, in particular with regards to research publication. It remains one of the main goals for each one of us, and we feel that our progress was too slow due to time constraints and limited skills. As shown in Table 2, most of our work is still not published. Another desired professional skill that needs further attention is proposal writing for independent research projects. We feel the need to work more intensely on these aspects of professional skills advancement:

“*Personally, I felt that although I had a PhD, my experience in publication was very limited and would have liked more support in this area from my supervisors to achieve this through specialised supervision for my first one and/or joint publication. In addition, I would have appreciated to develop skills in the area of research proposal writing. I felt these aspects of the PDRF need to improve for each fellow to be supported based on their needs and interests.*” (Post-doc A)

## Discussion

The findings section has highlighted several skills and capacities that we developed through the CHESAI PDRF as well as challenges encountered in the process. Our experiences speak to the PDRF process itself and the type and level of capacity development acquired. In a typical PDRF, the purpose is to build capacity of emerging researchers through mentorship to develop skills such as publication, networking, proposal writing and research grant management among others [42]. However, with the CHESAI PDRF, it was unique in that we did not have the specific background in HPSR although we had some relevant knowledge. The emergent nature of the HPSR field would not have been suitable to the structured PDRF required in the natural sciences, for instance [43]. Therefore, this created some challenges of lack of clear sense of direction and a feeling that expectations were not being met because, in the beginning, it was difficult to grasp the concept of an emergent and flexible PDRF approach. However, these feelings and experiences of being at loss in the beginning of a post-doc were not unique to us as CHESAI post-docs. Others have had similar experiences even in a well-structured programme as revealed in a survey of post-doctoral researchers’ experiences by Science Careers. One post-doc reported that, when she first started her post-doc, she felt intimidated and could not communicate as much as she eventually did afterwards and this experience acted as a barrier to her work progress [44].

A key element of the CHESAI PDRF was the apprenticeship aspect which allowed for experiential learning and was suited to the nature of the PDRF. Lansang and Dennis [45] report that a combination of short- and long-term strategies targeted at individuals, institutions and countries are vital for the development and sustainability of health research. One of the approaches is hands-on training on on-going research and mentorship programmes, applied in the form of apprenticeship programmes. These are considered relatively quick quantifiable outputs for human resources capacity development [45]. Both of these strategies have been applied in the CHESAI PDRFs, as discussed in the findings, whereby we were attached to projects through which we gained practical experience of researcher–practitioner engagement and we were involved in teaching and student co-supervision alongside our mentors in the form of apprenticeship. Cole et al. [25] also identified mentorship as crucial for capacity strengthening in health research.

Some authors have written about capacity building limitations related to the work environment, especially in LMICs, including a lack of competent institutional leaders, insufficient funds for research and salaries, inadequate infrastructure, inequitable access to scientific and technical information, and lack of active engagement with the research community [2, 37, 38]. However, we did not experience these challenges. Instead, we had very positive encounters with regards to the work environment. Our experiences show how relevant these institutional capacities are in supporting capacity development in both the short- and long-term. We also reflected on the possibilities we had for engaging in various activities, including research projects, access to information through journal clubs and bimonthly meetings. These are a demonstration of the capacities both at institutional and leadership levels that created opportunities for learning.

The need for the development of a critical mass of researchers in HPSR has also been highlighted by a number of authors [46–48]. The CHESAI PDRF contributed to this need through the recruitment of four post-docs, as presented in the Case study description. The fact that we come from four different countries in sub-Saharan Africa and with different but related backgrounds creates synergy among us to work together as a team in writing joint papers and sharing knowledge, both tacit and from our professional backgrounds. This is a positive step towards developing that critical mass of research competencies in HPSR. This has the potential for ripple effects through our various engagements towards enhancing the research agenda for HPSR in Africa beyond our post-doctoral fellowship. The idea of critical mass was also identified as a key factor by a team of African Emerging Leaders in their reflections on lessons learnt for future health policy and systems research and analysis and development programmes. They indicated that training in HPSR through nomination by employing organisations offers potential advantage of addressing succession planning and development of a critical mass of staff with HPSR skills [49].

Some of these authors have also emphasised the need for a plan on how to achieve capacity development, including commitment and effective leadership, as well as specific outputs for the researchers [46, 48]. Although these were not explicitly outlined in the CHESAI PDRF, we still managed to make a lot of progress in acquiring various skills, and were able to account for our time over the period of our engagement with tangible outputs such as conference presentations, participation in CHESAI activities, student co-supervision and examination, teaching, and paper writing among others. Since the CHESAI PDRF is still under way, we still anticipate more opportunities that will enable continued learning to further deepen our understanding of HPSR, through continued engagement in the CHESAI activities and development of specific professional skills such as critical writing, graduate student supervision, grant proposal writing and peer review processes. The mentoring relationships and collective writing process among ourselves as post-docs is a significant way of strengthening capacity development. Cole et al. [50], in their review of indicators for measuring capacity strengthening, identified similar issues and categorised them into output and outcome indicators. For instance, number of trainers having mentors, number of meetings/workshops attended by trainers, and quality of training, among others, were identified as output indicators, while research skills, published research work, number of grantees working as senior researchers, development and sustained research collaborations, trainers retaining to active and independent research in LMICs were identified as outcome indicators [50]. Some of these indicators apply in our post-doc experiences, whereby we had attendances in conferences, workshops, bimonthly meetings and journal clubs, writing retreats, and other fora as output indicators as well as all having mentors. However, since we are still in the post-doc programme, it is rather difficult to assess outcome indicators. There is one exception, where the PDRF who finished her 2-year appointment managed to establish a Centre for Health Policy and Systems Research in her country, of which she will be head after finalising her post-doc appointment. She will also hold the position of lecturer at her former employer – a university institution. An update to this paper in a year’s time would provide more insights into outcome indicators of this PDRF initiative after all the four post-docs complete their fellowship period.

Networking as an important aspect for career development as well as strengthening capacity for health research was given high priority in CHESAI through the South to South collaborations in the form of expert residents and special funding to conferences for post-docs and other CHESAI members to engage at various levels. In their assessment, Lenter et al. [51], concluded that networking can foster long-term professional relationships and enable exchange of knowledge, resources and mutual support among researchers with common interests. This is also evident in our CHESAI experience through exchange of knowledge among CHESAI members during journal clubs, bimonthly meeting writing retreats as well as the visiting expert residents and practitioner sabbaticals. An example of the one outcome is the collaborative research work we are currently involved in with one of the CHESAI resident experts.

Notwithstanding the positive achievement of the CHESAI PDRF, as noted in the findings section, there are a few shortcomings that are worth highlighting. First, the fact that the programme was flexible with no specific targets created a situation where we spent a lot of time in the beginning trying to figure out our place in the system, and to understand what it is we were supposed to be doing. Therefore, having some form of structure with certain priorities and deadlines would be helpful. Secondly, the lack of clarity in terms of the core skills and competencies required as an HPS researcher to progress to the next level after the PDRF to become an independent researcher, is also a challenge. The African Health Policy and Systems Research and Analysis (HPSR + A) future leaders programme participants identified some of these competencies based on their experiences. These include (1) personal skills such as communication and listening skills, patience, empathy and many more; (2) writing skills; (3) project management; (4) networking skills; (5) knowledge and understanding of HPSR + A; (6) teaching strategies; (7) understanding the health system; and (8) research skills [49]. However, most of these competencies have been achieved in our CHESAI post-doc as expressed in the findings section but not in a systematic way. For instance, one important aspect – project management, which includes grant proposal writing – was not successfully achieved but it is one of the critical skills for our future career development in HPSR. This may impact on the outcome indicators such as retaining to active and independent research work.

## Conclusion

Reflecting on the CHESAI PDRF has provided room to understand some of the critical issues on which capacity building in HPSR needs to focus. As a pioneering PDRF capacity initiative in the field, there is much that the HPSR community could learn from, improve and build upon. This reflection paper has shown that PDRF is a useful approach to capacity development in the field of HPSR, if post-docs are provided with the necessary space for learning, reflection and mentorship. Creating a good balance between theory and practice is essential in producing skills that have multilevel applications, and useful both in theory and practice. Therefore, a successful capacity development programme using PDRF in any field including HPSR requires certain fundamental facets. These include effective leadership, dedicated and supportive supervisors/mentors, a conducive environment for research and other activities with essential infrastructure and technology, embeddedness in community of practice, opportunities for networking, a modest financial package, flexibility, and spaces for reflection. All these are necessary preconditions for attaining a certain level of skills and competencies, and for developing the capacity of post-docs to work in and manage the challenges and complexities in the field of their specialisation. More academic institutions in Africa therefore need to adopt innovative and flexible support for emerging leaders, researchers and practitioners to strengthen our health systems.

## Abbreviations

CHESAI: Collaboration for Health Systems Analysis and Innovation
HPSR: health policy and systems research
PDRF: post-doctoral research fellowship

## Footnotes

1
http://hpsa-africa.org.

## References

[1] Sheikh K, Gilson L, Agyepong IA, Hanson K, Ssengooba F, Bennett S (2011). Building the field of health policy and systems research: framing the questions. PLoS Med.

[2] Bennett S, Agyepong IA, Sheikh K, Hanson K, Ssengooba F, Gilson L (2011). Building the field of health policy and systems research: an agenda for action. PLoS Med.

[3] Orgill M. (Blog post) Health Policy and Planning Debated. London School of Hygiene and Tropical Medicine. 2013. http://blogs.lshtm.ac.uk/hppdebated/2013/10/02/contemplating-capacity-development-for-health-policy-and-systems-research-in-africa/#sthash.IjAOgDao.dpuf. Accessed 12 May 2016.

[4] Gilson L (2012). Health Policy and Systems Research: A Methodology Reader.

[5] De Savigny D, Adam T (2009). Systems Thinking for Health Systems Strengthening.

[6] Mills A (2011). Health policy and systems research: defining the terrain; identifying the methods. Health Policy Plan.

[7] Block Gonzalez MA, Mills A (2003). Assessing capacity for health policy and systems research in low and middle income countries. Health Res Policy Sys.

[8] Adam T, Ahmad S, Bigdeli M, Ghaffar A, Røttingen J-A (2011). Trends in health policy and systems research over the past decade: still too little capacity in low-income countries. PLoS One.

[9] Mirzoev T, Le G, Green A, Orgill M, Komba A, Esena RK (2014). Assessment of capacity for Health Policy and Systems Research and Analysis in seven African universities: results from the CHEPSAA project. Health Policy Plan.

[10] Le G, Mirzoev T, Orgill M, Erasmus E, Lehmann U, Okeyo S (2014). A new methodology for assessing health policy and systems research and analysis capacity in African universities. Health Res Policy Sys.

[11] Potter C, Brough R (2004). Systemic capacity building: a hierarchy of needs. Health Policy Plann.

[12] Green A, Bennett S (2007). Sound Choices Enhancing Capacity for Evidence-Informed Health Policy.

[13] Bennett S, Paina L, Kim C, Agyepongi I, Chunharas S, McIntyre D et al. What must be done to enhance capacity for Health Systems Research? Background paper for the global symposium on health systems research, 16–19 November 2010, Montreux, Switzerland. http://healthsystemsresearch.org/hsr2010/images/stories/4enhance_capacity.pdf.

[14] Drotar D, Cortina S, Crosby LE, Hommel KA, Modi AC, Pai AL (2014). Competency-based postdoctoral research training for clinical psychologists: An example and implications. Train Educ Prof Psychol.

[15] Felisberti FM, Sear R (2014). Postdoctoral researchers in the UK: a snapshot at factors affecting their research output. PLoS One.

[16] Martinez A, Epstein C, Parsad A, Whittaker K (2012). Developing international research collaborations among Postdoctoral Fellows: Key Findings from the Evaluation of NSF’s International Research Fellowship Program.

[17] Stiers W, Barisa M, Stucky K, Pawlowski C, Van Tubbergen M, Turner AP (2015). Guidelines for competency development and measurement in rehabilitation psychology postdoctoral training. Rehabil Psychol.

[18] Hill ST, Hoffer TB, Golladay MJ. Plans for postdoctoral research appointments among recent U.S. doctorate recipients: InfoBrief. National Science Foundation; 2004.

[19] Su X (2014). Rank advancement in academia: what are the roles of postdoctoral training?. J High Educ.

[20] Berman J, Juniper TP, Thomson C (2008). Reconceptualising post-PhD research pathways: A model to create new postdoctoral positions and improve the quality of postdoctoral training in Australia. Aust Univ Rev.

[21] Enders J (2005). Border crossings: Research training, knowledge dissemination and the transformation of academic work. High Educ.

[22] Chen S, McAlpine L, Amundsen C (2015). Postdoctoral positions as preparation for desired careers: a narrative approach to understanding postdoctoral experience. High Educ Res Dev.

[23] McAlpine L, Amundsen C (2013). Early career researcher challenges: substantive and methods-based insights. Stud Contin Educ.

[24] Martinez A, Epstein CS, Pasad A (2016). Developing internationally engaged scientists and engineers: The effectiveness of an international postdoctoral fellowship program. Res Eval.

[25] Cole DC, Johnson N, Mejia R, McCullough H, Turcotte-Tremblay A, Barnoya J, Luco SF (2015). Mentoring health researchers globally: Diverse experiences, programmes, challenges and responses. Global Public Health.

[26] Melin G (2005). The dark side of mobility: Negative experiences of doing a postdoc period abroad. Res Eval.

[27] Stephan P, Ma J (2005). The increased frequency and duration of the postdoctorate career stage. Am Econ Rev.

[28] Gibbs G (1988). Learning by doing: A guide to teaching and learning methods.

[29] Yin RK (2009). Case study research: Design and methods.

[30] Gardner F (2014). Being critically reflective: engaging in holistic practice.

[31] Srivastava P, Hopwood N. A practical iterative framework for qualitative data analysis. Int J Qual Methods. 2009;8(1):Srivastava.

[32] Bassett R, Mills AJ, Durepos G, Wiebe E (2010). Iterative Data Analysis. Encyclopedia of Case Study Research.

[33] Hogg MK, Maclaren P (2008). Rhetorical issues in writing interpretivist consumer research. Qual Mark Res Int J.

[34] Mays N, Pope C. Qualitative research: Rigour and qualitative research. BMJ. 1995;311:109–122. 10.1136/bmj.311.6997.109 PMC2550154 7613363

[35] Aronson J (1994). A pragmatic view of thematic analysis. Qual Rep.

[36] Braun V, Clarke V (2006). Using thematic analysis in psychology. Qual Res Psychol.

[37] Baxter P, Jack S (2008). Qualitative case study methodology: study design and implementation for novice researchers. Qual Rep.

[38] Kolb A, Kolb B, Armstrong SJ, Fukami CV (2009). Experiential Learning Theory: A Dynamic, Holistic Approach to Management Learning, Education and Development. The SAGE Handbook of Management Learning, Education and Development.

[39] Bates I, Phillips R, Martin-Peprah R, Kibiki G, Gaye O, Phiri K (2011). Assessing and strengthening African universities’ capacity for doctoral programmes. PLoS Med.

[40] Adedokun B, Nyasulu P, Maseko F, Adedini S, Akinyemi J, Afolabi S (2014). Sharing perspectives and experiences of doctoral fellows in the first cohort of Consortium for Advanced Research Training in Africa. Glob Health Action.

[41] Kitto SC, Chesters J, Grbich C (2008). Quality in qualitative research: Criteria for authors and assessors in the submission and assessment of qualitative research articles for the Medical Journal of Australia. MJA.

[42] Pelham P (2015). Doing Postdoctoral Work — Should I?.

[43] Fonseca-Kelly Z, Operario DJ, Finger LD, Baucum II AJ. The Role of Post docs, PIs, and Institutions in Training Future Scientists. 2010. http://www.asbmb.org/asbmbtoday/asbmbtoday_article.aspx?id=9198. Accessed on 23 Oct 2015.

[44] Bonetta L. The Postdoc Experience: Not Always What You Expect. Science AAAS. Available on https://www.sciencemag.org/careers/features/2008/08/postdoc-experience-not-always-what-you-expect. Accessed on 22 Oct 2015.

[45] Lansang M, Dennis R (2004). Building capacity in health research in the developing world. Bull World Health Organ.

[46] Gyapong JO, Ofori-Adeji D (2006). Capacity Building for Relevant Health Research in Developing Countries.

[47] Hyder AA, Akhter T, Qayyum A (2003). Capacity development for health research in Pakistan: the effects of doctoral training. Health Policy Plan.

[48] Whitworth JAG, Kokwaro G, Kinyanjui S, Snewin VA, Tanner M, Walport M (2008). Strengthening capacity for health research in Africa. Lancet.

[49] CHEPSAA final networking meeting. Activities and outputs. 2015. http://www.slideshare.net/hpsa_africa/chepsaa-final-networking-meeting-setting-the-scene-framing-the-meeting. Accessed 14 Dec 2016.

[50] Cole DC, Boyd A, Aslanyan G, Bates I (2014). Indicators for tracking programmes to strengthen health research capacity in lower – and middle – income countries: a qualitative synthesis. Health Res Policy Sys.

[51] Lenters LM, Cole DC, Godoy-Ruiz P (2014). Networking among young global health researchers through an intensive training approach: a mixed methods exploratory study. Health Policy Res Sys.

